# Use of Stem Cell Extracellular Vesicles as a “Holistic” Approach to CNS Repair

**DOI:** 10.3389/fcell.2020.00455

**Published:** 2020-06-10

**Authors:** Heather Branscome, Siddhartha Paul, Dezhong Yin, Nazira El-Hage, Emmanuel T. Agbottah, Mohammad Asad Zadeh, Lance A. Liotta, Fatah Kashanchi

**Affiliations:** ^1^Laboratory of Molecular Virology, School of Systems Biology, George Mason University, Manassas, VA, United States; ^2^American Type Culture Collection (ATCC), Manassas, VA, United States; ^3^American Type Culture Collection (ATCC) Cell Systems, Gaithersburg, MD, United States; ^4^Department of Immunology and Nano-Medicine, Herbert Wertheim College of Medicine, Florida International University, Miami, FL, United States; ^5^Center for Applied Proteomics and Molecular Medicine, George Mason University, Manassas, VA, United States

**Keywords:** central nervous system, mesenchymal stem cells, induced pluripotent stem cells, extracellular vesicles, exosomes

## Abstract

Neurodegeneration is a hallmark of many diseases and disorders of the central nervous system (CNS). High levels of neuroinflammation are often associated with irreparable damage to CNS cells due to the dysregulation of signaling cascades that are unable to restore a homeostatic balance. Due to the inherent complexity of the CNS, development of CNS-related therapeutics has met limited success. While stem cell therapy has been evaluated in the context of CNS repair, the mechanisms responsible for their functional properties have not been clearly defined. In recent years, there has been growing interest in the use of stem cell extracellular vesicles (EVs) for the treatment of various CNS pathologies as these vesicles are believed to mediate many of the functional effects associated with their donor stem cells. The potency of stem cell EVs is believed to be largely driven by their biological cargo which includes various types of RNAs, proteins, and cytokines. In this review, we describe the characteristic properties of stem cell EVs and summarize their reported neuroprotective and immunomodulatory functions. A special emphasis is placed on the identification of specific biological cargo, including proteins and non-coding RNA molecules, that have been found to be associated with stem cell EVs. Collectively, this review highlights the potential of stem cell EVs as an alternative to traditional stem cell therapy for the repair of cellular damage associated with diverse CNS pathologies.

## Introduction

The central nervous system (CNS) is regarded as the most complex system in the human body and its associated diseases and disorders represent leading causes of death and disability worldwide. These pathologies encompass a wide range of conditions ranging from neuro-degenerative diseases [e.g., Alzheimer’s disease (AD), Parkinson’s disease (PD), and multiple sclerosis], to brain cancer, ischemia, traumatic brain and spinal cord injury, and viral infections of the CNS (Gooch et al., 2017; Ben Abid et al., 2018; Erkkinen et al., 2018). Collectively, these pathologies represent a major burden to the global healthcare industry. As the incidence of neurodegenerative diseases rises in aging populations, this burden is expected to substantially increase due to the associated increases in life expectancy (Wyss-Coray, 2016; GBD 2016 Neurology Collaborators, 2019).

Neurodegeneration is a common feature among many CNS pathologies. In recent years the association between neuronal damage and neuroinflammation has received much attention as it is becoming increasingly evident that chronic and/or aberrant inflammation may contribute to the progression of many neurodegenerative disorders (Amor et al., 2010; Stephenson et al., 2018). Indeed, many of the cellular processes that are involved in the neurodegenerative cascade (e.g., apoptosis, necroptosis, autophagy, and demyelination) can be driven by inflammatory responses, and CNS-resident astrocytes and microglia are the predominant cell types which mediate these effects (Chen et al., 2016; Chitnis and Weiner, 2017). However, it is important to note that in addition to perpetuating damage, the immune system also plays a pivotal role in mediating both reparative and regenerative mechanisms (Stephenson et al., 2018). Therefore, further studies are warranted to delineate the underlying factors that control these responses.

Historically, therapeutics targeting the CNS have met limited success and have experienced higher than average failure rates relative to non-CNS therapeutics. These failures are attributed to multiple factors such as the extended timeframes and higher costs associated with CNS drug development, stringent regulatory procedures, challenges associated with drug delivery across the blood–brain-barrier (BBB) and, perhaps most importantly, an overall lack of comprehensive knowledge surrounding both normal brain physiology and CNS disease pathology (de Lange et al., 2017; Gribkoff and Kaczmarek, 2017; Danon et al., 2019). These gaps of knowledge underscore the importance for continued research and novel therapeutic strategies targeting the CNS.

During the last decade stem cells have emerged as potential therapeutic candidates for various CNS pathologies. This is largely due to their multipotent, neurotrophic, and immunomodulatory properties (Song et al., 2018). Specifically, mesenchymal stem cells (MSCs) represent a prominent source of stem cells which have been extensively studied for different biomedical applications. MSCs are non-hematopoietic stem cells which can be isolated from various tissues throughout the body [e.g., bone marrow (BM), adipose tissue, and amniotic fluid] and possess multilineage differentiation potential when cultured under the appropriate conditions (Chamberlain et al., 2007; Samsonraj et al., 2017). MSCs have demonstrated broad reparative and regenerative properties in various studies, including those relating to bone, cardiac, liver, lung, kidney, skin, and CNS which have been extensively reviewed elsewhere (Trounson and McDonald, 2015; Han et al., 2019). To further illustrate this point, a current search of clinicaltrials.gov using the search criteria of “mesenchymal stem cells” yields over 300 trials that are designated as active, recruiting, or enrolling.

With respect to MSCs, it should be noted that due to the heterogeneity of their origin these cells must meet a set of pre-defined criteria prior to their use in laboratory-based and pre-clinical studies. These criteria were published in 2006 by the Mesenchymal and Tissue Stem Cell Committee of the International Society for Cellular Therapy in an effort to standardize cell preparations among laboratories (Dominici et al., 2006). Specifically, the guidelines state that MSCs must demonstrate plastic-adherence in standard tissue culture flasks, exhibit specific expression of surface markers (e.g., positive expression of CD105, CD73, CD90 and negative expression of CD45, CD34, CD14, or CD11b, CD79a, or CD19, HLA class II), and possess the ability to differentiate into osteoblasts, adipocytes, and chondroblasts *in vitro*. Thus, by adhering to these guidelines, more meaningful comparisons of scientific data and experimental outcomes relating to MSCs can be achieved (Dominici et al., 2006).

Recent advances in biotechnology have further evolved the field of stem cell research. For example, the generation of induced pluripotent stem cells (iPSCs) has resulted in substantial progress to be made in the context of disease modeling, drug discovery, and cell-based therapy over the last several years (Shi et al., 2017). Utilizing specialized protocols, adult somatic cells can be genetically reprogrammed into iPSCs via the forced expression of key transcription factors (i.e., Oct3/4, Sox2, Klf4, and c-Myc) (Takahashi et al., 2007). Once generated, iPSCs display properties similar to those of embryonic stem cells (ESCs) and, therefore, have the ability to differentiate into cells of each of the three germ layers. This technology was first applied in 2006 by Takahashi and Yamanaka when they generated iPSCs from adult mouse cells; in 2007 these same researchers reported the successful reprogramming of human somatic cells into iPSCs (Takahashi and Yamanaka, 2006; Takahashi et al., 2007).

The therapeutic potential of iPSCs has been studied in conditions ranging from corneal, cartilage, and cardiac repair to immunotherapy (Jiang et al., 2014; Yoshida and Yamanaka, 2017; Castro-Viñuelas et al., 2018; Chakrabarty et al., 2018). With respect to the CNS, iPSCs have been highly regarded for their potential to model neurodegenerative diseases and this is largely attributed to their ability to differentiate into multiple different neuronal and glial lineages (Haston and Finkbeiner, 2016). To illustrate this point, iPSCs have been used to model Alzheimer’s, Parkinson’s, and Huntington’s diseases as well as several motor neuron diseases (Ebert et al., 2009; Israel et al., 2012; Juopperi et al., 2012; Burkhardt et al., 2013; Nihei et al., 2013; Woodard et al., 2014).

From a mechanistic viewpoint, the extracellular vesicles (EVs) that are released by stem cells are believed to mediate many of the multi-functional effects that are attributed to their donor stem cells (Phinney and Pittenger, 2017). EVs are nano-sized vesicles that are secreted by most, if not all, cell types and act as signaling messengers between cells. Broadly speaking, EVs can be classified based on their size and biogenesis, with exosomes and microvesicles (MVs) representing the two subtypes of EVs that are the most commonly studied (van Niel et al., 2018). Briefly, exosomes range from 50 to 150 nm in diameter and originate from the endocytic pathway. Exosomes are contained within multivesicular bodies (MVBs) and upon fusion of MVBs to the plasma membrane, exosomes are released into the extracellular space. In contrast, MVs can have diameters ranging from 50 to 1,000 nm and are shed directly from the plasma membrane via outward budding (Akers et al., 2013; Zaborowski et al., 2015; van Niel et al., 2018).

It is important to note that due to vesicle heterogeneity, lack of standardized nomenclature, and the various methods utilized for vesicle purification, it is challenging to effectively distinguish between exosomes and MVs. For these reasons we believe that much of the literature describing reparative exosomes may actually refer to a mixed population of vesicles ranging from 30 to 250+ nm in size, of which exosomes represent only a fraction of the total population. Additionally, it is worth noting that The International Society for Extracellular Vesicles (ISEV) endorses the generic term “extracellular vesicle,” and the MISEV2018 guidelines strongly urge authors to clearly describe the physical and biochemical characteristics of the vesicles used in their studies (Théry et al., 2018). For the purposes of this review and to maintain consistency in terminology we will use the generic term “EV” when referencing the work of others.

The biological cargo that is associated with EVs includes various types of RNAs and proteins. These molecules can be horizontally transferred to recipient cells and, thus, have the ability to significantly affect or alter cellular activity through interactions with intracellular targets and signaling pathways (Phinney and Pittenger, 2017). Numerous studies have begun to investigate the therapeutic potential of both MSC and iPSC EVs and their multi-functional effects on processes relating to cellular repair and regeneration have been extensively reviewed (Sabapathy and Kumar, 2016; Börger et al., 2017; Phinney and Pittenger, 2017; Keshtkar et al., 2018; Taheri et al., 2019; Zhao T. et al., 2019). Additionally, stem cell EVs are now being evaluated as potential therapeutic tools for CNS repair (Koniusz et al., 2016; Luarte et al., 2016; Galieva et al., 2019).

Here, we summarize recent literature surrounding the biochemical characterization and functional effects of stem cell EVs, specifically those that have been isolated from either MSCs, iPSCs, or iPSC derivatives, with a focus on studies relating to CNS repair. Collectively, this review highlights the potential of stem cell EVs as an alternative approach to traditional stem cell-based approaches for the repair of cellular damage associated with CNS pathologies.

## Biological Cargo Associated With Stem Cell EVs

The potency of stem cell EVs is believed to be driven by the various types of bioactive cargo associated with these vesicles. This cargo includes lipids, RNAs, proteins, and other important signaling molecules such as cytokines, chemokines, and interleukins (Deng H. et al., 2018). The identification of EV-associated cargo is critical from both a characterization and analytical perspective as this information can be used to generate reference standards and to better define and understand the functional attributes of EVs. As such, many studies have begun to extensively profile the molecular content of EVs derived from different sources of stem cells. Here, we discuss important studies relating to EV-associated cargo and highlight specific molecules that may be associated with regenerative properties.

### RNA Profiling of Stem Cell EVs

While it has been previously reported that stem cell EVs carry intact mRNA that can be transferred horizontally and translated in recipient cells (Ratajczak et al., 2006; Tomasoni et al., 2013; Ragni et al., 2017), most studies to date have focused on the non-coding RNA components. Despite not coding for protein, non-coding RNAs (ncRNAs), including both small non-coding (<200 nucleotides) and long non-coding (≥200 nucleotides) RNAs, play important regulatory roles in a number of physiological processes including those relating to the CNS (Mattick, 2009; Zhou et al., 2016; Wang et al., 2017). Thus, EV-associated ncRNAS can be regarded as mediators of *trans*- regulation with the potential to significantly alter the phenotype of recipient cells (Valadi et al., 2007; Fatima et al., 2017).

#### Small Non-coding RNAs

Small ncRNAs include microRNA (miRNA), piwi-interacting RNA, transfer RNA (tRNA), Y RNA (yRNA), small nuclear RNA (snRNA), and small nucleolar RNA (snoRNA), among others (Wang et al., 2017). Despite this diversity, miRNAs represent the most widely studied type of ncRNAs and it has been reported that between 30 and 80% of protein coding genes are regulated by miRNAs. This is primarily achieved through the complementary binding of miRNA to a target mRNA sequence, resulting in either mRNA degradation or translational repression (Filipowicz et al., 2008; Lu and Clark, 2012).

Several studies have comprehensively analyzed the small RNAomes of EVs from different sources of stem cells and compared them to those of their donor stem cells. The first in-depth sequencing of MSC EVs was performed by Baglio et al. (2015). This study demonstrated that EVs from different sources of MSCs have distinctly different RNA profiles relative to their donor cells. Whereas cellular samples had high abundance of miRNAs and snoRNAs, EVs were found to be enriched with small fragments (14–30 nucleotides) of tRNAs as well as small subsets of mature miRNA transcripts, which collectively represented less than 5% of the total RNAome (Baglio et al., 2015). This study was the first to suggest that ncRNAs are selectively packaged into MSC EVs, a finding that was previously reported in EVs from cancer cells and immune cells (Hessvik et al., 2012; Nolte-’t Hoen et al., 2012). Interestingly, while the exact function of EV-associated tRNAs are not well understood, it has been suggested that tRNAs may function in a manner similar to miRNA (Kumar et al., 2014). When the antisense complements to the most abundant tRNAs were evaluated the potential targets included proteins involved in the inflammatory pathway (Baglio et al., 2015). These findings warrant further investigation to determine if tRNAs could contribute to the immunomodulatory properties of stem cell EVs.

While there is no collective miRNA signature attributed to stem cell EVs, others have observed the findings described by Baglio et al. (2015) which suggested that the EV miRNA landscape is dominated by relatively few and high abundant transcripts. For example, the top miRNAs in different EV preparations have been found to represent between 40 and 79% of the total miRNA associated with stem cell EVs (Fang et al., 2016; Ferguson et al., 2018). Additionally, a study by Kaur et al. (2018) compared the miRNA landscape of EVs from both MSCs and pluripotent stem cells (PSCs). Distinct differences were observed between EVs isolated from each cell type; specifically, miRNAs relating to maintaining pluripotency and cellular differentiation were most abundant in EVs from PSCs while miRNAs associated with MSC EVs were found to regulate various processes relating to differentiation, cell survival, and immunomodulation (Kaur et al., 2018). While these studies are suggestive that certain ncRNAs may be selectively packaged into stem cell EVs, the factors that contribute to this packaging have not been defined. Given the variable factors that can influence cell culture it is highly likely that the microenvironment, as well as the differentiation potential of the donor cells, may affect the sorting of EV cargo. However, it is interesting to note that the addition of the sequence GGCU was previously reported to promote sorting of miRNAs into EVs, while another study found specific enrichment of 3′-uridylated miRNAs in EVs relative to their donor cells (Koppers-Lalic et al., 2014; Santangelo et al., 2016). Further studies are needed to examine the mechanisms by which this may occur.

Highly abundant miRNAs from MSC EVs have been found to regulate processes relating to both wound healing and angiogenesis. Fang et al. (2016) identified 4 highly expressed miRNAs in umbilical cord MSC EVs (miR-21, miR-23a, miR-125b, and miR-145) that suppressed the formation of myofibroblasts via inhibition of the transforming growth factor-β2/SMAD2 pathway in a model of wound healing. In a recent study of adipose derived MSC EVs, Mitchell et al. (2019) also identified 3 of these miRNAs (miR-23a, miR-125b, and miR-145) within the top 50. Also, highly abundant were several anti-inflammatory miRNAs from the miR-let7family which have been shown to reduce fibrosis, modulate macrophage polarization, and to target TLR-4 pro-inflammatory signaling (Banerjee et al., 2013; Teng et al., 2013; Ti et al., 2015; Wang et al., 2016; Mitchell et al., 2019).

Another extensive study revealed that the top 23 miRNAs from BM-MSC EVs targeted genes which control angiogenesis. Among these were miR-21, miR-1246, miR-23a-3p, and miR-130a-3p which have previously been shown to modulate angiogenesis through varying mechanisms including activation of Akt and ERK (miR-21), Smad signaling (miR-1246), targeting Sprouty2 and Sema6A (miR-23a-3p), and downregulation of anti-angiogenic homeobox genes (miR-130a-3p) (Chen and Gorski, 2008; Chhabra et al., 2010; Feng et al., 2014; Yamada et al., 2014; Ferguson et al., 2018). *In vitro*, miR-130a-3p enriched EVs promoted angiogenesis in HUVECs as measured by branch length, segment number, and total number of nodes and junctions. Moreover, treatment with miR-130a-3p EVs reduced the expression of anti-angiogenic homeobox gene HOXA5 by approximately 90% (Ferguson et al., 2018).

miRNA analysis has also been performed on EVs from iPSCs, although to a somewhat lesser extent relative to MSC EVs. Similar to what has been observed in MSC EVs, Bobis-Wozowicz et al. (2015) reported that iPSC EVs contained substantially less miRNA relative to their donor cells and that a fraction of the total miRNAs were highly abundant in these vesicles. Pathway analysis revealed that the 29 most abundant miRNAs targeted multiple genes in pathways relating to focal adhesion, cell cycle, apoptosis, signal transduction (e.g., Wnt, PI3K-Akt, and MAPK), VEGF signaling, and RIG-I receptor signaling, among others (Bobis-Wozowicz et al., 2015).

More recently, Povero et al. (2019) identified approximately 700 miRNAs in iPSC EVs; from these a total of 22 miRNAs were chosen for subsequent analysis based on their relative abundance. Several of the most highly expressed miRNAs, such as miR-92a-3p, miR-26a-5p, miR- 22-3p, and miR-486-5p, have been shown to mediate anti-fibrotic effects through various mechanisms (e.g., targeting of pro-fibrotic mediators, attenuation of fibroblast *trans*-differentiation and epithelial-mesenchymal transition, and suppression of cell proliferation) (Berschneider et al., 2014; Graham et al., 2014; Liang et al., 2014; Li et al., 2016). The anti-fibrotic effects of iPSC EVs were further evaluated in an *in vivo* mouse model of liver fibrosis. Notably, EVs significantly decreased the expression of several pro-fibrogenic genes (e.g., αSMA, Collagen Iα1, and TIMP), decreased collagen deposition, and decreased activation of hepatic stellate cells; highly suggesting that the candidate miRNAs identified above may be partially responsible for mediating these effects (Povero et al., 2019). While the focus of this particular study was on liver fibrosis, it is important to note that fibrosis is highly associated with many chronic inflammatory diseases, and dysregulation of this process can lead to significant tissue damage and organ malfunction (Wynn and Ramalingam, 2012). Therefore, the observed anti-fibrotic effects of stem cell EVs could have broad application to many different pathologies.

Taken together, the studies described above are significant because they represent high-level, comprehensive analyses that reveal both the similarity and the diversity that exists among RNA cargo associated with stem cell EVs.

#### Long Non-coding RNAs

Compared to the small ncRNAs, studies relating to lncRNAs associated with stem cell EVs are relatively scarce; perhaps due to the relative complexity of their molecular mechanisms, their heterogeneity, and their poorly conserved nature (Beermann et al., 2016). However, recent research has found the lncRNA MALAT1 to be associated with stem cell EVs and there is mounting evidence that this lncRNA may regulate regenerative processes. Cooper et al. (2018) demonstrated a potential role for MALAT1 (adipose MSC EVs) in wound healing. Using an electric cell- substrate impedance sensing assay, cellular migration of human dermal fibroblasts significantly increased upon treatment with MALAT1-containing EVs whereas depletion of MALAT1 from EVs failed to enhance cellular migration (Cooper et al., 2018). MALAT1 (umbilical cord MSC EVs) was also found to prevent aging-induced cardiac dysfunction (Zhu et al., 2019). Here, treatment of cardiomyocytes with MALAT1-containing EVs decreased NFκB activity and resulted in reduced levels of p-p65. Additionally, decreases of inflammatory marker TNFα as well as aging marker p21 were observed at both the mRNA and protein level. Thus, suggesting that that the anti-aging effects of MSC EVs may be mediated through a novel MALAT1/NFκB/TNFα pathway (Zhu et al., 2019).

In the context of the CNS, MALAT1 may be responsible for mediating reparative functions. El Bassit et al. (2017) first described a neuroprotective role for this lncRNA in which MALAT1 (adipose MSC EVs) mediated splicing of the pro-survival protein kinase C δII, which promoted neuronal proliferation and survival *in vitro*. Similarly, *in vivo* studies also demonstrated potential neuroprotective effects of MALAT1 (adipose MSC EVs) as measured by improvement in motor impairment and reduction of lesion volume in a mouse model of traumatic brain injury (TBI). Here, analysis of gene expression patterns revealed that a number of the genes altered in response to treatment with stem cell EVs containing MALAT1 were related to the inflammatory response, signal transduction, cell survival and apoptosis. Moreover, this pattern was not observed in response to treatment with stem cell EVs that had been depleted of MALAT1 (Patel et al., 2018).

An additional role by which lncRNAs may act as miRNA sponges has also been suggested (Paraskevopoulou and Hatzigeorgiou, 2016). Yang et al. (2019) recently described a similar role for MALAT1 (BM-MSC EVs) in the context of alleviating osteoporosis. Through binding to miR-34c, MALAT1 promoted the expression of a key protein required for osteogenic differentiation, SATB2, which is an expected target of miR-34c. Furthermore, treatment of human osteoblasts with MALAT1-containing EVs resulted in increased expression of Runx2 and ATF4 which are two proteins important for osteogenic differentiation. Thus, the pro-osteogenic functions of MSCs EVs were attributed to the sponging of miR-34c via MALAT1 (Yang et al., 2019).

Similarly, Zhao W. et al. (2019) found that the lncRNA PVT1 was enriched in EVs from BM-MSCs. EV-associated PVT1 increased/stabilized the expression of oncogenic protein ERG, which correlated to increased proliferation and migration of osteosarcoma cells *in vitro* and promoted tumor growth and metastasis *in vivo*. Interestingly, EVs derived from PVT1 knockdown cells negated these effects, thereby highlighting their potential therapeutic application for osteosarcoma. In addition to the direct interaction between PVT1 and ERG, it was confirmed that PVT1 also promoted ERG expression through the sponging of miR-183-5p (Zhao W. et al., 2019).

It has also been suggested that in addition to sponging miRNAs, exosomal lncRNAs may serve as molecular sponges for other molecules including RNA-binding proteins (RBPs) (Kim et al., 2017). This potentially novel and relatively unexplored function of EV-associated lncRNA deserves further attention as this could have significant downstream effects on numerous cellular processes. Therefore, we believe further research into the lncRNA landscape of stem cell EVs is needed not only to explore this uncharacterized field but to also complement the pre-existing literature surrounding EV-associated small ncRNAs.

#### Circular RNAs

Circular RNAs (circRNAs) represent a unique class of non-coding RNAs that are gaining increased attention. Briefly, circRNAs arise from back splicing and covalent linking of 3′ and 5′ splice sites; thus resulting in highly stable, closed loop structures (Lasda and Parker, 2016). While this area of research is relatively novel, there is mounting evidence that circRNAs act as post- transcriptional regulators (e.g., miRNA sponges) in various biological processes and, in particular, may be involved in the progression of cancer (Zhong et al., 2018; Bach et al., 2019). Previous studies have found that circRNAs are expressed from multiple genomic locations (e.g., coding and non-coding exons, intergenic regions) and, moreover, that they demonstrate both tissue and developmental- specific expression patterns (Memczak et al., 2013; Maass et al., 2017; Xia et al., 2017; Mahmoudi and Cairns, 2019).

A study by Li et al. (2015) was the first to show enrichment of circRNAs in EVs. Here, it was reported that the expression of circRNA in EVs was at least two-folder higher than in donor cells and that the ratio of circRNA to linear RNA in EVs was approximately sixfold higher relative to donor cells. While the above observations were made in a panel of various cancer cell lines, these authors subsequently detected over 1,000 intact and stable circRNAs in serum EVs obtained from 3 healthy donors (Bao et al., 2015; Li et al., 2015).

Another recent study also found that circRNAs co-precipitated with EVs from three different cell lines (Hela, 293T, and U-2 OS) and RT-qPCR revealed that the ratios of circRNAs to their linear RNA counterparts were higher in EVs relative to donor cells (Lasda and Parker, 2016). While the underlying mechanisms remain unknown, these studies highly suggest that circRNAs could be selectively incorporated into EVs as a mechanism of clearance from the donor cell. For a more thorough review of the biogenesis and effects of EV-associated circRNAs we direct the reader to a recent review published by Wang et al. (2019).

Although circRNAs have been associated with metastatic phenotypes, it stands to reason that these RNAs could also modulate cellular repair. Along these lines, Sun et al. (2018) performed an extensive characterization of circRNAs to assess their potential involvement in the process of MSC-mediated repair of endometrial stromal cells (ESCs). Here, over 5,000 circRNAs were found to be significantly upregulated after MSC co-culture and results from enrichment analysis revealed the most significant pathways and biological processes to be related to DNA repair or cell proliferation (Sun et al., 2018).

In 2019, another study identified circFOXP1 as a specific marker of MSCs from multiple sources (e.g., cord blood, BM, adipose tissue, and Wharton’s jelly). Interestingly, knockdown of circFOXP1 in MSCs reduced both cellular proliferation and expression of characteristic MSC cell surface markers. *In vivo*, circFOXP1 knockdown was also associated with reduction of bone repair in a fracture/osteotomy model, thereby suggesting a potential functional role for circFOXP1 in maintaining the identity and reparative properties of MSCs (Cherubini et al., 2019). While the topic of EVs was outside of the scope of these experiments, studies of this nature nonetheless set an important precedent for future investigation into the potential functional effects of stem cell EV- associated circRNA.

### Proteomic Profiling of Stem Cell EVs

Equally important, from both a characterization and functional perspective, is the proteomic content of stem cell EVs. While EV biogenesis can occur via both ESCRT-dependent and ESCRT-independent mechanisms, ESCRT components and their accessory proteins such as Alix, TSG101, HSC70 and HSP90β are commonly regarded as EV marker proteins independent of their cell source (Doyle and Wang, 2019). Other proteins that are commonly associated with EVs include transport proteins (Rab GTPases and annexins), signal transduction factors (kinases), integrins (CD81, CD63, and CD9), metabolic enzymes, and cytoskeletal proteins (Simons and Raposo, 2009; Chaput and Théry, 2011). Here we will discuss other important protein cargo that has been found to be associated with stem cell EVs.

The initial characterization of the BM-MSC EV proteome was performed by Kim et al. (2012) and identified 730 proteins with high confidence. Through extensive functional analysis, several candidate proteins expected to mediate potential therapeutic effects were identified from this study. These included surface receptors (e.g., PDGFRB and EGFR), cell adhesion molecules (e.g., fibronectin and integrins), and multiple proteins involved in signaling pathways relating to MSC self-renewal and differentiation (e.g., Wnt, TGFβ, and RAS-MAPK pathways) (Kim et al., 2012).

Lai et al. (2012) also performed an early proteomic analysis on MSC EVs and through functional clustering found that 32 biological processes were over-represented. Among them were processes related to EV biogenesis, cellular motility, inflammation, and proteolysis. Interestingly, this study revealed that all subunits of the 20S proteasome were present in the MSC EVs and, furthermore, that this complex was found to be enzymatically active *in vitro*. *In vivo*, 20S proteasome functionality was assayed in a mouse model of myocardial reperfusion injury and treatment with EVs significantly lowered the levels of misfolded proteins (Lai et al., 2012). These results point toward a potential mechanism by which MSC EVs may exert a protective effect through the proteolytic degradation of damaged proteins.

More recently, using a high-resolution mass spectrometry approach, Anderson et al. (2016) evaluated the proteome of BM-MSC EVs from multiple different donors and detected a total of 580 membrane associated proteins within each sample. Among those detected were ATPases, EGFRs, G proteins, RAB family members, various protein kinases, as well as several known EV- and MSC-specific markers. Through the generation of an angiogenesis interactome network, robust protein interactions between PDGFR, EGFR, and NFκB proteins were revealed. Furthermore, EVs derived from MSCs exposed to ischemic conditions were enriched in angiogenic signaling proteins (e.g., EGF, FGF, and PDGF), suggesting that under stressful conditions cells may release EVs containing proangiogenic factors to facilitate localized tissue repair. When these EVs were applied to HUVECs that had been treated with an NFκB inhibitor they failed to induce tubular formation, supporting the theory that NFκB signaling may be required to mediate the angiogenic potential of MSC EVs (Anderson et al., 2016).

In further support of a potential protein-mediated angiogenic effect, it was found that EVs from umbilical cord MSCs could directly deliver VEFG to epithelial cells in an *in vivo* model of renal ischemia. The authors attributed this transfer of VEGF to the corresponding increase in renal angiogenesis and capillary density in EV-treated animals (Zou et al., 2016). Additionally, we recently detected VEGF and FGF-2, out of a total of 40 cytokines, as the two highest iPSC EV- associated cytokines. In an *in vitro* angiogenesis co-culture assay, significant increases in tubular length were observed in response to treatment with iPSC EVs (Branscome et al., 2019). A recent review has also highlighted other pro-angiogenic proteins that have been associated with MSC EVs; these include TGF-β1, IL-8, thrombopoietin, and MMPs (Merino-González et al., 2016).

Others have also identified EV proteomic cargo that may contribute to tissue repair and wound regeneration. Shabbir et al. (2015) found that BM-MSC EVs could enhance cell growth and migration in both normal and diabetic wound fibroblasts *in vitro*. Through further analysis, it was shown that exposure to MSC EVs resulted in activation of AKT, ERK, and STAT3 signaling pathways and, moreover, significantly induced the expression of several cell cycle and growth factor (e.g., HGF, IGF1, and NGF) genes (Shabbir et al., 2015).

Bakhtyar et al. (2018) performed a similar experiment to evaluate the effect of MSC EVs (acellular Wharton’s jelly from umbilical cord) on cell viability and migration. Treatment with EVs significantly enhanced cell viability and migration *in vitro*; interestingly, when EVs were lysed this effect was diminished, thereby suggesting that intact EVs are required to exert downstream effects. While proteomic analysis of EVs identified a number of proteins associated with wound healing (e.g., actin aortic smooth muscle, vimentin, fibrillin, and fibronectin), the proteinase inhibitor alpha-2-macroglobulin (α2M) was one of the most highly abundant proteins. *In vitro*, the addition of exogenous α2M significantly increased cellular viability and migration, thereby highlighting a novel role by which α2M in stem cell EVs may contribute to the wound healing process via the inhibition of proteinases (Bakhtyar et al., 2018).

Liu S. et al. (2019) observed that both MSC and iPSC EVs reduced reactive oxygen species (ROS) and alleviated the phenotypes associated with aging using *in vitro* models of cellular senescence. Specifically, in response to stem cell EVs, the number of cells expressing senescence- associated beta-galactosidase, gene expression of p21 and p53, and production of cytokines IL-6 and IL-1A were all lower. Moreover, there was reduced expression of γ-H2AX protein, which is associated with DNA damage. Analysis of the proteomes identified approximately 1,135 proteins that were common among MSC and iPSC EVs and, among these several antioxidant enzymes belonging to the peroxiredoxin (PRDX) family were abundant. Notably, treatment of senescent cells with purified plasma EVs, which had undetectable levels of PRDX proteins, had minimal effect on the aging phenotype relative to stem cell EVs. Thus, it was proposed that PRDX enzymes delivered by stem cell EVs may play a role in mitigating oxidative stress and repairing age-associated damage (Liu S. et al., 2019). While other studies have also reported similar findings relating to the effect of stem cell EVs on aging cells (Kulkarni et al., 2018; Oh et al., 2018; Zhu et al., 2019), this study provided a novel proteomic insight as to how this may be mediated.

Kumar et al. (2019) profiled the proteomic composition of EVs from placental MSCs and found proteins with functions related to extracellular matrix (e.g., collagen, fibronectin, and laminin), MMPs (e.g., MMP1, MMP2, and various ADAM proteins), and numerous signaling molecules (e.g., HGF, galectin-1, PDGF, and TGF-β). Biological processes related to the MSC proteome, as identified by STRING, included wound healing, extracellular matrix organization and disassembly, cell adhesion, neurogenesis, and axonogenesis. *In vitro*, MSC EVs were found to exert a neuroprotective effect on apoptotic cells as measured by increases in neurite outgrowth and branching points. Further analysis supported a potential role for galectin-1, which was present on the surface of EVs, in mediating these functions as pre-incubation of EVs with anti-galectin-1 partially abrogated the neuroprotective effects (Kumar et al., 2019).

Finally, a recent study compared various size EVs, including exomeres, from a variety of cancer cell lines (Zhang et al., 2019). Exomeres represent the most recently discovered and smallest type of EV, with an estimated size of ≤50 nm. Here, these colleagues found that exomeres were enriched in proteins relating to metabolism, glycan processing, and mTORC1 signaling; confirming observations from a previous study (Zhang et al., 2018) of exomeres. Interestingly, exomeres were also enriched in Argonaute (Ago 1, Ago 2, and Ago 3) proteins and contained a higher amount of small RNAs relative to EVs, indicating that miRNAs may be enriched in exomeres (Zhang et al., 2019). Further studies are needed to see if this data reproduces in stem cell EVs.

### EV-Associated Lipids and Other Metabolites

Lipids also play an important role in EV biogenesis and are involved in several steps relating to EV fate and bioactivity. For example, it has been shown that EV-associated lipids (e.g., phosphatidylserine and lysophosphatidylcholine) are required to bridge intercellular communication (Freeman et al., 2010) and, furthermore, can serve as chemoattractants for lymphocytes (Radu et al., 2004) and induce maturation of dendritic cells (Perrin-Cocon et al., 2004; Deng H. et al., 2018). Previous research has also revealed that MSC EVs contain several critical glycolytic enzymes, namely glyceraldehyde-3-phosphate dehydrogenase, phosphoglycerate kinase, lactate dehydrogenase A, aldolase A, enolases, and pyruvate kinase m2 isoform; many of which are among the top 25 EV-associated proteins according to ExoCarta (Lai et al., 2012; Toh et al., 2018).

Showalter et al. (2019) also found that EVs from primed MSCs, which had been cultured in serum free medium under hypoxic conditions, displayed altered lipidomic and metabolic profiles. Specifically, EVs had a higher content of lipid membrane components (e.g., phosphatidyl-ethanolamine phospholipids, ceramide, and lysophosphatidylcholines) and were packaged with metabolites linked to carbohydrate, amino acid, and nucleoside metabolism. Moreover, several distinct metabolites with immunomodulatory functions (e.g., adenosine, arginine, aspartic acid, cholesterol, glutamine, nicotinamide, palmitic acid, and isoleucine) were also detected in EVs (Showalter et al., 2019). This data suggests that priming of cells has the potential to reprogram the metabolic profile of EVs and that this altered metabolic content may enhance their functional capabilities in recipient cells.

The lipid content of EVs may also contribute to their observed pro- or anti-inflammatory functional effects. For example, Toh et al. (2018) found that EV subpopulations can be differentiated based upon affinity for membrane lipid-binding ligands, namely cholera toxin B chain (CTB), annexin V (AV) and Shiga toxin B chain (ST), which bind to GM1 ganglioside, phosphatidylserine, and globotriaosylceramide. Along these lines, it may be possible to customize the mode of EV purification to better allow for characterization of EV binding and delivery of cargo into recipient cells.

Taken together, the results outlined in this section highlight the complex and diverse nature of the biological cargo that is associated with EVs from different sources of stem cells. Despite this complexity, EVs from stem cells share the common ability to exert protective and reparative effects in a wide range of cellular contexts. Due to the heterogeneous nature of EV cargo, it is not likely that one single candidate molecule is responsible for eliciting a therapeutically relevant biological response. Rather, EV-associated cargos are likely to work in a complementary manner to synergistically mediate the pleiotropic effects attributed to stem cell EVs. This synergy may hold relevance from a therapeutic perspective considering the fact that previous CNS trials based on single cytokines have been unsuccessful due to factors relating to half-life, inability to access CNS, and low distribution (Drago et al., 2013). Due to their rich composition and their ability to cross the blood brain barrier, stem cell EVs may overcome these challenges. However, the kinetics underlying the interactions between EV cargo and their target pathways have yet to be fully defined. A more thorough understanding of the contribution of EV RNAs and proteins is therefore necessary and will be absolutely required in order to advance EV-based therapeutics.

## Stem Cell EVs for CNS Repair

Recent years have experienced an abundant interest in the use of stem cell EVs, particularly those derived from MSCs, to treat various CNS pathologies. Several *in vitro* and *in vivo* studies have demonstrated that these vesicles have the potential to regulate various cellular processes, such as neurogenesis, angiogenesis, and neuroinflammation, that are critical for the repair of damaged CNS cells. In the following sections we highlight and discuss many of these key findings. Tables 1, 2 summarize the respective *in vitro* and *in vivo* studies of the selected references.

**TABLE 1:** *In vitro* studies using stem cell EVs for CNS-related repair.

Study	Vesicle source	Vesicle type	Vesicle cargo	Recipient cell type	Functional effect
Kumar et al., 2019	Placental MSCs; human	Exosomes	Galectin-1	SH-SY5Y; human neuroblastoma cell line; treated with staurosporine to induce apoptosis	Increased circuitry length, branch points, and tube length; increased cell number
Ching et al., 2018	Adipose MSCs differentiated into Schwann cell-like phenotype; rat	Exosomes	miR-18a, miR-182, miR-21, miR-222; Gap43 and Tau mRNA	NG108-15; mouse neuroblastoma cell line	Increased neurite outgrowth
Yuan et al., 2019	BM-MSCs; human	Exosomes	Fibronectin	SH-SY5Y; human neuroblastoma cell line	Induced proliferation; promoted secretion of mitogenic and neurotrophic factors
Bodart-Santos et al., 2019	Wharton’s jelly MSCs; human	EVs	Catalase	Primary hippocampal cells; rat; exposed to AβOs	Reduced oxidative stress; prevented AβO-induced synaptic damage
Bonafede et al., 2016	Adipose MSCs; mouse	Exosomes	Not studied	NSC34; mouse motoneuron-like cell line expressing ALS mutations and challenged with oxidative stress	Rescued cell viability; reduced apoptosis
Reis et al., 2018	BM-MSCs; human	EVs	miR-21-5p	Monocyte-derived DCs; human	Decreased antigen uptake by immature DCs; modulated DC maturation and secretion of cytokines; reduced DC migration
Deng M. et al., 2018	ESCs and ESC-derived NPCs	Exosomes	Not studied	ESC-derived neurons; human; oxygen–glucose deprivation	Increased neuronal survival; reduced apoptosis; reduced expression of pro-inflammatory factors; attenuated mTOR signaling

*EVs, extracellular vesicles; AβOs, amyloid beta oligomers; ALS, amyotrophic lateral sclerosis; MSCs, mesenchymal stem cells; BM, bone marrow; DCs, dendritic cells; ESCs, embryonic stem cells; NPCs, neural progenitor cells; mTOR, mammalian target of rapamycin.*

**TABLE 2:** *In vivo* studies using stem cell EVs for CNS-related repair.

Study	Disease model	Vesicle source	Vesicle type	Route of administration	Outcomes
Huang et al., 2017	SCI	BM-MSCs; rat	Exosomes	Tail vein injection	Reduced lesion size; reduced apoptosis and inflammation; promoted angiogenesis
Li et al., 2018	SCI	BM-MSCs over-expressing miR-133b; rat	Exosomes	Tail vein injection	Improved functional recovery; decreased lesion cavity; preserved NeuN+ neurons; enhanced axonal outgrowth
Ruppert et al., 2018	SCI	BM-MSCs; human	EVs	Tail vein injection	Improved locomotor recovery and mechanical sensitivity threshold; attenuated neuroinflammation
Liu W. et al., 2019	SCI	BM-MSCs; rat	Exosomes	Tail vein injection	Reduced apoptosis and formation of glial scars; promoted axon regeneration; suppressed activation of microglia and A1 neurotoxic astrocytes
Lu et al., 2019	SCI	BM-MSCs; rat	EVs	Tail vein injection	Improved motor recovery; reduced neuronal death; preserved BSCB integrity; increased BSCB pericyte coverage
Zhao C. et al., 2019	SCI	BM-MSCs; rat	Exosomes	Tail vein injection	Improved motor function; attenuated complement activation; inhibited NFκB activation
Ni et al., 2019	TBI	BM-MSCs; rat	Exosomes	Retro-orbital injection	Reduced lesion area; reduced apoptosis and inflammation; downregulated M1 phenotype and upregulated M2 phenotype
Sun et al., 2019	TBI	NSCs; human	EVs	Tail vein injection	Enhanced migration of NSCs to site of injury; increased VEGFR2 expression; improved motor function
Williams et al., 2019	TBI	BM-MSCs; human	Exosomes	Intravenous injection	Reduced injury severity; improved neurocognitive recovery
Drommelschmidt et al., 2017	Perinatal brain injury	BM-MSCs; human	EVs	Intraperitoneal injection	Reduced cellular degeneration and apoptosis; prevented reactive gliosis; improved myelination and cognitive deficits; restored white matter integrity
Thomi et al., 2019	Perinatal brain injury	Wharton’s jelly MSCs; human	Exosomes	Intranasal administration	Reduced expression of pro-inflammatory molecules; reduced microgliosis
Chang et al., 2019	Sepsis syndrome	Adipose MSCs; rat	Exosomes	Intravenous injection	Reduced infiltration of inflammatory cells in brain tissue; reduced expression of brain damage biomarkers, apoptotic markers, inflammatory biomarkers, and oxidative stress biomarkers
Ding et al., 2018	AD	UC-MSCs; human	Exosomes	Tail vein injection	Improved spatial learning and memory; reduced Aβ plaques; increased expression of Aβ-degrading enzymes; alleviated neuroinflammation
Elia et al., 2019	AD	BM-MSCs; mouse	EVs	Intracerebral injection	Reduced Aβ plaque area; reduced plaque solidity; reduced formation of dystrophic neurites
Reza-Zaldivar et al., 2019	AD	MSCs; human	Exosomes	Stereotaxic surgery	Improved cognitive functions and increased neurogenesis
Long et al., 2017	Status Epilepticus	BM-MSCs; human	Exosomes	Intranasal	Reduced expression of pro-inflammatory cytokines; reduced activation of microglia; reduced neuronal loss; reduced loss of inhibitory interneurons; preserved reelin+ neurons
Xin et al., 2013a	Stroke	BM-MSCs; rat	Exosomes	Tail vein injection	Improved functional recovery; increased synaptic plasticity, neurite remodeling, neurogenesis, and angiogenesis
Xin et al., 2013b	Stroke	BM-MSCs; rat	Exosomes	Tail vein injection	Increased synaptic plasticity and neurite remodeling; promoted neurogenesis and angiogenesis
Doeppner et al., 2015	Stroke	BM-MSCs; human	EVs	Intravenous injection	Improved neurological recovery; increased long-term neuronal density; increased angioneurogenesis; reversed lymphopenia
Xin et al., 2017b	Stroke	BM-MSCs over-expressing miR-133b; rat	Exosomes	Intra-arterial injection	Improved neurological outcome; increased axonal density and synaptic plasticity
Xin et al., 2017a	Stroke	BM-MSCs over-expressing miR-17-92; rats	Exosomes	Intravenous injection	Improved functional recovery; increased neurite remodeling; increased neurogenesis and oligodendrogenesis
Otero-Ortega et al., 2018	Stroke	Adipose MSCs; rat	EVs	Tail vein injection	Promoted axonal sprouting; increased expression of oligodendrocyte-associated markers; promoted myelin restoration
Webb et al., 2018	Stroke	NSCs; human	EVs	Intravenous injection	Decreased lesion volume and cerebral swelling; preserved diffusivity and white matter integrity; increased exploratory behavior and motor activity; improved temporal and spatial gait parameters
Deng et al., 2019	Stroke	BM-MSCs; mouse	Exosomes	Injection (not specific)	Reduced neuronal apoptosis; reduced inflammation
Baulch et al., 2016	Irradiation	NSCs; human	MVs	Intrahippocampal transplantation	Improved cognitive function; reduced activation of microglia; preserved structure of hippocampal neurons
Smith et al., 2020	Irradiation	NSCs; human	EVs	Intrahippocampal transplantation	Preserved dendritic complexity; protected neurons against degradation; increased dendritic spine density; reduced activation of microglia

*EVs, extracellular vesicles; MVs, microvesicles; MSCs, mesenchymal stem cells; NSCs, neural stem cells; BM, bone marrow; UC, umbilical cord; TBI, traumatic brain injury; SCI, spinal cord injury; BSCB, blood spinal cord barrier; NSCs, neural stem cells; Aβ, amyloid beta.*

### Stem Cell EV-Mediated Repair *in vitro*

Several recent studies have reported the neuroprotective effects of stem cell EVs in various different recipient cell types. Using an *in vitro* apoptotic model of the human neuroblastoma cell line SHSY5Y, Kumar et al. (2019) found that treatment with EVs from placental MSCs resulted in a significant increase in cell number, circuitry length, and branch points. Analysis of EV proteomic data revealed several pathways (e.g., adhesion, extracellular matrix receptor interaction, PI3K-Akt, Rap1, and axon guidance) that were regulated (Kumar et al., 2019). Using the same neuroblastoma cell line, another study found that exposure to MSC EVs that had been generated under primed conditions (reduced serum, 1% oxygen) induced cellular proliferation and promoted secretion of several proteins with mitogenic and neurotrophic functions (e.g., HGF, VEGF, TGFβ1/β3, FGF-7, BDNF, GDNF, and PDGF-AA) (Yuan et al., 2019). Extensive proteomic analysis confirmed that EVs were packaged with over 700 extracellular proteins associated with proliferation; among these fibronectin was the most abundant. Subsequent assays using inhibitors against both Akt and fibronectin signaling attenuated the mitogenic effects of EVs, thus demonstrating that cellular proliferation was mediated in a fibronectin-dependent manner (Yuan et al., 2019). The results from this study also highlighted how EV biogenesis may be potentiated to enhance their functional effects.

Ching et al. (2018) found that EVs isolated from adipose stem cells which had been differentiated into a Schwann cell-like phenotype could promote neurite outgrowth in the mouse neuroblastoma cell line NG108-15. Analysis of EV RNA identified four miRNAs (miR-18a, miR-182, miR-21, and miR-222) which have previously been shown to be enriched in axons, promote Schwann cell proliferation, promote neurite outgrowth and nerve regeneration, and inhibit neuronal apoptosis (Strickland et al., 2011; Zhou et al., 2012; Iyer et al., 2014; Zhou et al., 2015). Additionally, Gap43 and Tau-coding mRNAs, which encode proteins important for neurite growth and regeneration, were upregulated in EVs (Frey et al., 2000; Ching et al., 2018). Treatment of NG108-15 cells with EVs fluorescently labeled with an RNA-specific dye revealed that EV-associated RNA was internalized and distributed among the cell body and nerve axons. Interestingly, exposure of EVs to UV light significantly reduced their neuritogenic properties. Overall this data suggests that both EV miRNAs and mRNAs may be important mediators of their functional effects (Ching et al., 2018).

Bodart-Santos et al. (2019) recently reported the neuroprotective effects of MSC EVs in response to damage by amyloid-β oligomers (AβOs). Using rat primary hippocampal cultures, the authors found that EVs rescued AβO-induced oxidative stress by reducing the level of ROS to that of the control and, moreover, that this effect was mediated by catalase contained within the EVs. Since AβOs are known to reduce the levels of pre- and post-synaptic marker proteins (Lourenco et al., 2013; Brito-Moreira et al., 2017; Batista et al., 2018) the effect of EVs on synaptic density was next examined. Notably, a 22-h incubation with EVs was able to completely eliminate synaptic damage and, again, this was found to be mediated in a catalase-dependent manner. Interestingly, fluorescent labeling of EVs revealed that they were primarily taken up by astrocytes in the hippocampal cultures, thereby highlighting the potential of stem cell EVs to mediate the function of astrocytes in AD (Bodart-Santos et al., 2019). MSC EVs have also demonstrated neuroprotective effects in an *in vitro* model of amyotrophic lateral sclerosis (ALS). Here, Bonafede et al. (2016) utilized different constructs of the motoneuron-like NSC-34 cell line engineered to express 3 human ALS mutations [SOD1(G93A), SOD1(G37R), and SOD1(A4V)]. Apoptosis was induced via exposure to hydrogen peroxide; double staining with AO/PI revealed that treatment with EVs restored cellular viability to that of the control in each cell line tested (Bonafede et al., 2016).

Deng M. et al. (2018) recently evaluated the effects of EVs from different cell types including ESCs and ESC-derived neural progenitor cells. Addition of these EVs to oxygen–glucose deprived (OGD) human neurons resulted in increased survival rate, reduction of apoptosis, and attenuation of OGD-induced expression of pro-inflammatory factors (e.g., COX-2, iNOS, and TNFα). Further analysis indicated that EVs also exerted effects on the mammalian target of rapamycin signaling pathway as measured by decreased expression of p-AMPK and increased expression of p-PI3K p85 and p-Akt (Deng M. et al., 2018).

From an immunological perspective, Reis et al. (2018) were the first to perform a comprehensive analysis of the immunomodulatory effects MSC EVs on the function of human dendritic cells (DCs) *in vitro*. When DCs were exposed to EVs antigen uptake was impaired, expression of CD83, CD38, CD80 maturation/activation markers were reduced, pro-inflammatory cytokine secretion was decreased, and anti-inflammatory cytokine production was increased. Furthermore, the expression of CCR7, a known regulator of DC migration, was also reduced and DCs exhibited a significant reduction in their ability to migrate to the CCR7 ligand CCL21 after treatment with EVs. When EV miRNA content was analyzed, several miRNAs with known effects on DC function and maturation (e.g., miR-21-5p, miR-142-3p, miR-223-3p, and miR-126-3p) were within the top 10. Among these miR-21-5p was found to target CCR7, and DCs engineered to overexpress this miRNA partially recapitulated the effects associated with MSC EV treatment (Reis et al., 2018). Therefore, EV-associated miRNA may be responsible for modulating DC phenotypes.

### Stem Cell EV-Mediated Repair *in vivo*

#### SCI, TBI, and Other Brain Injury

The neuroprotective effects of stem cell EVs on various types of brain injury has been highly studied. In the context of spinal cord injury (SCI), Liu W. et al. (2019) performed a comprehensive analysis and found that MSC EVs attenuated neuronal death, reduced formation of glial scars, suppressed microglia and neurotoxic astrocyte (A1) activation, reduced the production of pro-inflammatory cytokines (e.g., TNFα, IL-1β, and IL-6), and promoted axon regeneration. Here, the suppression of microglial and A1 activation was postulated to reduce neuronal apoptosis and contribute to the overall neuronal repair necessary for the recovery of functional behavior in rats (Liu W. et al., 2019). These results were consistent with an earlier study by Huang et al. (2017) who also found that administration of MSC EVs improved hindlimb locomotor activity of rats and reduced lesion size post-SCI. Treatment with EVs also reduced apoptosis, decreased levels of TNFα and IL1β, and promoted angiogenesis; again indicating that EVs could ameliorate the effects associated with brain trauma and promote tissue repair and functional recovery (Huang et al., 2017).

Ruppert et al. (2018) have also reported beneficial effects of MSC EVs in a rat model of SCI. Specifically, locomotor recovery improved, mechanical hypersensitivity increased, and neuroinflammation was attenuated in response to EVs from both unstimulated and TNFa/IFN-γ stimulated MSCs. Notably, these effects were observed despite administration of treatment 3 h post-injury, a timeframe which is expected to have a more realistic translation to real-world application (Ruppert et al., 2018). Lu et al. (2019) recently examined the effects of EVs on the blood-spinal cord barrier (BSCB) following SCI. Here, treatment with EVs was associated with a reduction of BSCB permeability and an increased coverage of PDGFRβ positive pericytes. Mechanistically, the functional effects of EVs were attributed to a downregulation of NFκB p65 signaling (Lu et al., 2019). In another study, Zhao C. et al. (2019) investigated the effects of MSC EVs on the complement system after SCI. Proteomic analysis revealed downregulation of several complement proteins in EV-treated animals and RT-PCR confirmed signification reductions in C1q, Cfh, C3, C4b, C6, C5, Mbl, Cfp complement mRNA. Additionally, EVs downregulated p-p65 and p-IκBα, thereby inhibiting the SCI-induced activation of NFκB (Zhao C. et al., 2019).

Interestingly, Li et al. (2018) demonstrated that MSC EVs could be engineered with enhanced levels of miR-133b, a miRNA which has previously been associated with neuroprotective properties (Xin et al., 2012, 2013b; Lu et al., 2015; Niu et al., 2016). Administration of miR-133b EVs promoted functional recovery, decreased lesion volume, and promoted axonal outgrowth after SCI relative to control EVs. Treatment with EVs also resulted in decreased expression of RhoA, a known target of miR-133b that is associated with neuronal death (Wu et al., 2017) and increased phosphorylation of ERK1/2, STAT3, and CREB (Li et al., 2018). These results suggest that the neuroprotective effects of miR-133b EVs may be mediated through the activation of pathways relating to cell survival and axon regeneration.

Ni et al. (2019) studied the functional properties of MSC EVs during the early stages post-TBI. Here, retro-orbital injection of EVs not only improved behavioral performance but also attenuated apoptosis and reduced the expression of IL-1β and TNFα in the injured cortex. Immunofluorescence revealed a decrease in iNOS+/Iba1+ cells and an increase in CD206+/Iba1+ cells 3 days post-injury, suggesting that EVs modulated the polarization of microglia/macrophages from the classic (M1) to the alternative (M2) phenotype (Ni et al., 2019). Sun et al. (2019) also evaluated the functional and histological effects of human neural stem cell EVs in a rat model TBI. They found that EV-treated animals exhibited reduced tissue loss, increased presence of neural stem cells around the injury site, increased expression of vascular endothelial growth factor receptor 2 (VEGFR2), and demonstrated enhanced motor function. Interestingly, the authors noted that these effects appeared to be stronger in male rats, a finding that warrants further investigation into the potential sex-dependent effects of EVs (Sun et al., 2019). Williams et al. (2019) also observed protective effects of MSC EVs in a clinically relevant large animal model of TBI. Assessment of neurocognitive functions strongly suggested that EV treatment attenuated the severity of injury and permitted faster recovery (Williams et al., 2019).

Thomi et al. (2019) also found that MSC EVs decreased production of pro-inflammatory cytokines TNFα, IL-1β and reduced microgliosis in a rat model of perinatal brain injury. Through subsequent studies it was found that the anti-inflammatory effects of EVs were partially mediated via interference with the TLR-4/CD14 signaling cascade (Thomi et al., 2019). In a similar study Drommelschmidt et al. (2017) also observed that MSC EVs reduced apoptosis and cellular degeneration in both the cortex and white matter, prevented reactive gliosis of microglia and astrocytes, and improved myelination. Additionally, EV treatment improved cognitive deficits (e.g., memory function and exploratory activity) and improved white matter integrity after inflammatory brain injury (Drommelschmidt et al., 2017). Thus, stem cell EVs may contribute to functional recovery through the modulation of brain microstructure and neuro- inflammation.

Lastly, Chang et al. (2019) evaluated the therapeutic potential of MSC EVs against sepsis- syndrome induced brain damage. Results from this study revealed several neuroprotective effects in response to EV treatment; these included a reduced infiltration of inflammatory cells (e.g., F4/80+, CD14+, MMP-9+, and GFAP+ cells), reduced expression of brain damage biomarkers (e.g., AQP4 and γ-H2AX), and reduced expression of apoptotic and oxidative stress biomarkers. However, the authors noted the most important finding to be the potential EV-mediated suppression of a DAMP component (HMGB1) as well as several of its downstream inflammatory markers (e.g., TLR-2, TLR-4, MYD88, IL-1β, TNFα, NFκB, and MMP-9) (Chang et al., 2019). Since the brain is highly susceptible to damage associated with the inflammatory response, these results suggest that stem cell EVs may represent a novel therapeutic platform for sepsis syndrome.

#### Neurodegenerative Disorders

The therapeutic potential of MSC EVs has also been evaluated in several animal models of neurodegeneration. Reza-Zaldivar et al. (2019) found that MSC EVs significantly improved cognitive function and neurogenesis in a beta amyloid 1-42 induced model of AD. Notably, 28 days after administration, the outcomes of EV-treatment animals were comparable to those of the normal controls as assessed by Morris water maze and novel object recognition tests as well as immunofluorescent analysis of PSA-NCAM+ and DCX+ cells in the subventricular zone, which indicated stimulation of neurogenesis (Reza-Zaldivar et al., 2019). Ding et al. (2018) also found that MSC EVs were functional in a double-transgenic mouse model of AD. Here, administration of EVs improved spatial learning and memory, lowered the number of amyloid beta (Aβ) plaques in the cortex and hippocampus, and increased the levels of Aβ-Degrading Enzymes Insulin-degrading enzyme (IDE) and Neprilysin (NEP). Results from RT-qPCR showed increased gene expression of M2 microglia markers YM-1, Arg-1, MRC1, and ELISA confirmed reduced levels of pro-inflammatory cytokines IL-1β and TNFα in both the peripheral blood and brains of EV-treated animals, suggesting that EVs could alleviate neuroinflammation through the alternative activation of microglia (Ding et al., 2018). Elia et al. (2019) also investigated the effects of MSC EVs during the early stages of AD using the APPswe-PS1dE9 double transgenic mouse model. EVs strongly reduced the Aβ plaque area, plaque solidity, and plaque density in different regions of the brain, leading the authors to propose that EVs not only facilitated disaggregation of pre-existing plaques but also slowed the formation of new ones. Immunostaining further revealed a decrease of Smi31-32 positive neurofilaments in the brains of treated animals, indicating that EVs reduced the formation of dystrophic neurites around Aβ plaques (Elia et al., 2019).

Neuroprotective effects of MSC EVs have also been observed in a mouse model of status epilepticus (SE). Notably, labeling of EVs revealed robust localization to neurons (frontoparietal cortex and the dorsal hippocampus) and microglia (rostral regions of cortex) after SE (Long et al., 2017). EV treatment reduced the expression of 7 pro-inflammatory cytokines [TNFα, IL-1β, MCP-1, stem cell factor (SCF), MIP-1α, GM-CSF, and IL-12], increased the expression of 5 anti-inflammatory cytokines/growth factors (IL-10, G-CSF, PDGFβ, IL-6, and IL-2), and prevented aberrant neurogenesis in the hippocampus. Moreover, EVs decreased microglia activation in the hippocampus, increased expression of NeuN neurons, and reduced the loss of PV, SS, and NPY GABAergic interneurons in the hippocampus. Behavioral studies confirmed that EV-treated animals demonstrated enhanced performance in object location test, novel object recognition test, and pattern separation test; collectively suggesting that stem cell EVs may repress the neuroinflammation, neurodegeneration, and cognitive impairments associated with SE (Long et al., 2017).

#### Stroke

Xin et al. (2013a) evaluated the effects of MSC EVs on functional recovery and neuronal remodeling in a rat model of stroke. These studies were the first to show that EV treatment increased synaptic plasticity, neurite remodeling, neurogenesis, and angiogenesis in the ischemic boundary zone (Xin et al., 2013a). In separate studies, these colleagues subsequently found that engineering of MSC EVs to overexpress either miR-133b (Xin et al., 2017b) or miR- 17-92 cluster (Xin et al., 2017a) further amplified many of these outcomes. Interestingly, it was shown that the miR-17-92 over-expressing EVs decreased the levels of PTEN, leading to activation of downstream PI3K/Akt/mTOR signaling and subsequent inactivation of GSK-3β. Thus, the therapeutic benefits of these EVs were attributed to direct targeting of the PTEN axis (Xin et al., 2017a).

In addition to the neuroprotective and neuroregenerative properties listed above, Doeppner et al. (2015) also observed that MSC EVs exerted effects on the peripheral immune response in a mouse model of ischemic stroke. Specifically, EVs were found to deactivate dendritic cells and to reverse lymphopenia at 6 days post-injury as measured by absolute counts of B- cells, NK cells, and T cells. Due to the fact that MSC EVs are generally regarded for their anti- inflammatory actions, these results were particularly important because they demonstrated that MSC EVs do not exacerbate post-stroke immunosuppression (Doeppner et al., 2015).

Mesenchymal stem cells EVs have also been shown to contribute to the repair of white matter in a rat model of subcortical stroke. As shown by Otero-Ortega, a single administration of MSC EVs not only improved functional recovery but also enhanced axonal sprouting, promoted myelin restoration, and increased the expression of several markers related to oligodendrocyte maturation (e.g., CNP-ase, A2B5, and MOG) (Otero-Ortega et al., 2018). Deng et al. (2019) recently showed that MSC EVs over- expressing miR-138-5 further reduced neuronal apoptosis and expression of IL-6, IL-1β, and TNFα relative to control MSC EVs in a middle cerebral artery occlusion mouse model. These effects were believed to be largely mediated via miR-138-5 targeting of LCN2, a protein which is highly associated with brain injury and inflammation (Deng et al., 2019).

Using a large animal model of ischemic stroke, Webb et al. (2018) studied the effects of human neural stem cell EVs at both the functional and tissue level. EV treatment decreased lesion volume and cerebral swelling and, furthermore, examination of tissue 84 days post- injury revealed preservation of white matter integrity. Additionally, EV-treated animals displayed increased exploratory behavior and motor activity and exhibited improved temporal and spatial gait parameters (Webb et al., 2018). Thus, the results from these experiments provided clinically relevant end points for the therapeutic application of stem cell EVs against stroke. Many of the results described in this subsection have been further discussed in a recent review by Doeppner et al. (2018).

#### Irradiation

Different studies have also pointed toward an EV-mediated repair in tissue that has been damaged by irradiation. This is particularly important considering that cancer patients exposed to cranial irradiation often develop debilitating cognitive dysfunction (Meyers, 2000; Butler et al., 2006). The first evidence of this was provided by Baulch et al. (2016) who performed cranial grafting of EVs from human neural stem cells (hNSCs) into the hippocampus of rat subjects that had been exposed to 10 Gy of irradiation. These authors found that 1 month after transplantation, EV-treated animals exhibited significant improvements in three different cognitive tasks (novel object recognition, novel place recognition, and object in place). While exposure to irradiation severely compromised neuronal complexity and architecture, grafting of EVs reduced the damage to hippocampal neurons as visualized by Golgi staining and quantification of different structural parameters (e.g., dendritic length, volume, and capacity). Moreover, EVs significantly reduced the amount of activated microglia in brain regions that were proximal and distal to the site of engraftment, thereby suggesting that EVs may spare the brain from irradiation-induced cognitive deficits (Baulch et al., 2016).

In a more recent follow-up study, these effects were further explored using a similar experimental design (Smith et al., 2020). Here, treatment with EVs restored dendritic spine density and preserved dendritic complexity. These results were attributed to the EV-mediated restoration of the neurotrophic factor glial cell line-derived neurotrophic factor (GDNF), which was detected in higher levels in the ipsilateral and contralateral regions of EV-treated animals. Further experiments also revealed that EV treatment attenuated the irradiation-induced increase of the postsynaptic scaffolding protein PSD-95, a protein which has strongly correlated to the cognitive reductions associated with irradiation (Smith et al., 2020). Taken together, these studies strongly suggest that stem cell EVs may have the potential to mitigate the detrimental and debilitating side effects that are associated with cranial radiation. A more comprehensive review of this innovative topic was recently published by Leavitt et al. (2019).

#### Engineered EVs

The production of “designer” EVs with enhanced therapeutic potential have also been reported. For example, EVs from MSCs that were stimulated with IFNγ have proven to be effective in repairing and reversing cellular damage in an experimental autoimmune encephalomyelitis (EAE) mouse model. Here, Riazifar et al. (2019) demonstrated that administration of EVs yielded several beneficial effects such as reduced demyelination, decreased neuroinflammation, induction of CD4+ CD25+ FOXP3+ regulatory T cells (Tregs), in addition to the observed functional Improvements. RNA sequencing further revealed that these EVs were enriched with anti-inflammatory RNAs, including Indoleamine 2,3-dioxygenases 1 and Thymosin beta 10 mRNA as well as miR-146b, and proteomic analysis of EVs also identified a number of neuroprotective proteins (e.g., Galectin-1, Aggrecan, Testican 1, and Periostin) (Riazifar et al., 2019). Thus, this study demonstrated that the priming of donor stem cells may produce EVs with enhanced functional ability.

Interestingly, Kojima et al. (2018) have generated an EXOtic device which simultaneously enhances EV biogenesis and RNA packaging into EVs as well as EV secretion and delivery of mRNA into recipient cells. Implantation of EXOtic into a mouse model of PD resulted in the production of EVs that could successfully deliver therapeutic catalase mRNA into the brain which, in turn, reduced neuroinflammation and attenuated neurotoxicity. While these results were obtained using a construct generated with human embryonic kidney (HEK) cells, the authors also noted that the EXOtic device had demonstrated functionality in MSCs (Kojima et al., 2018). Overall, this system is of particular interest since the implantation of modified producer cells can constitutively produce drug-containing EVs, which may offer a long-term therapeutic approach for conditions that require repeated or long-term treatment.

**FIGURE 1:**
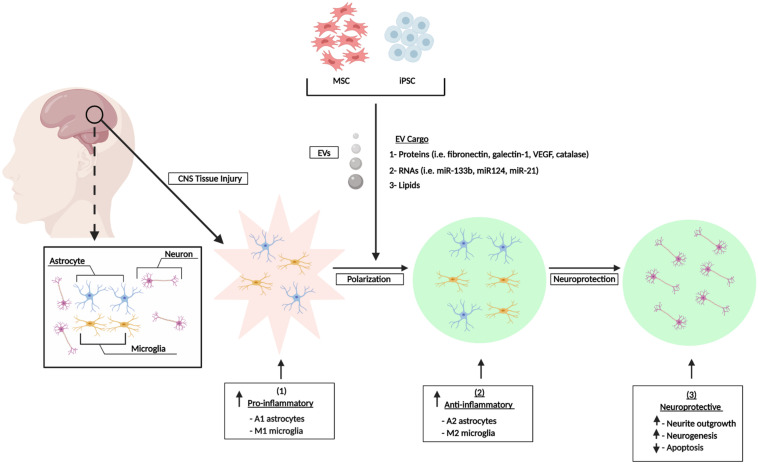
Stem cell EV-mediated repair of CNS damage *in vitro*. CNS injury induces an inflammatory response characterized by increased levels of pro-inflammatory astrocytes (A1) and microglia (M1). Stem cells secrete a heterogenous population of EVs which promote the polarization of astrocytes and microglia into their A2 and M2 anti-inflammatory phenotypes, respectively. This confers an overall neuroprotective phenotype. Figure adapted from Katsuda and Ochiya (2015) and Deng M. et al. (2018).

**FIGURE 2:**
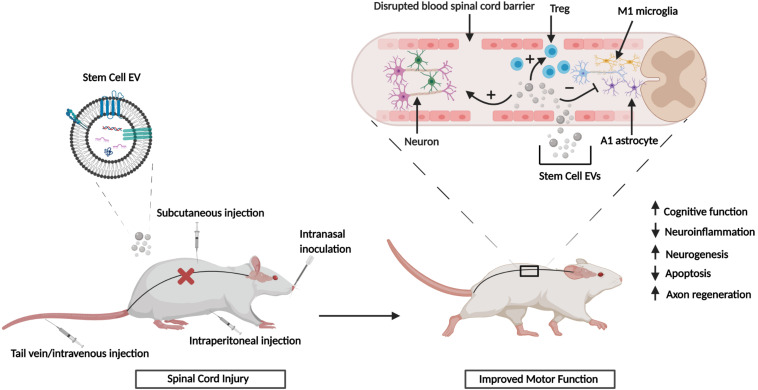
Stem cell EV-mediated CNS repair *in vivo*. SCI results in the disruption of the blood spinal cord barrier. Upon injury, stem cell EVs can be administered via several different routes. Treatment with stem cell EVs is characterized by both functional and histological improvements. Figure adapted from Guo et al. (2019) and Riazifar et al. (2019).

In summary the *in vitro* and *in vivo* studies referenced above have shown that EVs from different sources of MSCs can exert potent, multifunctional effects in various scenarios of CNS damage. The observed effects include modulation of neuroinflammation via polarization of M1 and A1 astrocytes to their alternative M2/A2 phenotypes and mediation of other neuroprotective effects that are characterized by reduced cell death, enhanced neurogenesis and neurite outgrowth, and reduction of synaptic damage (Figure 1). Collectively, these outcomes may contribute to the partial repair or reversal of associated functional and neurological impairments. Furthermore, the performance of numerous animal studies has shown that the *in vitro* effects of stem cell EVs can be effectively recapitulated *in vivo* and this has been showcased in various animal models of CNS injury. Figure 2 summarizes the general effects associated with the *in vivo* administration of stem cell EVs in the general context of SCI, although it is important to note that the neuroprotective effects of stem cell EVs are conserved across a wide range of pathological conditions. While these results are largely observed in response to MSC EVs, our previous research has suggested that EVs from human iPSCs may also promote the recovery of certain CNS cell types, namely astrocytes and monocyte-derived macrophages, under radiation-induced stress. Moreover, we have observed distinct differences in EV-associated cytokines and lncRNAs among MSC and iPSC EVs (Branscome et al., 2019). This is a new and exciting area of research which deserves further attention to better characterize the effects of iPSC EVs both *in vitro* and *in vivo* in the context of CNS repair. Additionally, these types of studies will be critical to determine if the functional effects conveyed by MSC EVs can be reproduced using EVs from different sources of stem cells and, furthermore, to distinguish any potential differences in mechanisms which may exist between stem cell EVs.

## Pro- and Anti-Tumorigenic Effects of Stem Cell EVs

It is also worth noting, as pointed out in a recent review, that there have been contrary publications on the effects of EVs that support either cancer progression or suppression (Katsuda and Ochiya, 2015). For instance, several studies have provided evidence that MSC EVs may support and promote a cancer phenotype (Zhu et al., 2012; Lin et al., 2013; Munoz et al., 2013; Roccaro et al., 2013; Du et al., 2014), whereas others have demonstrated an overall anti- tumorigenic effect of MSC EVs (Fonsato et al., 2012; Bruno et al., 2013; Katakowski et al., 2013; Wu et al., 2013). Although these studies are well-designed and controlled, it is important to note that there are differences in the recipient cell types used and this may impact the ultimate phenotype that is observed upon uptake of EVs. For example, as described by Wu et al. (2013), MSC EVs attenuated growth of bladder cancer cells, while Du et al. (2014) found that MSC EVs promoted the growth of renal cancer cells. At this point, it is hard to accurately assess whether these observed phenotypes may be impacted by the EV donor cells (due to age, sex, genetic differences, etc.) or if they are related to the specific nature of the recipient cancer cells. Additionally, future studies should also evaluate check point proteins such as p53, retinoblastoma protein, Cdks and cyclins, all of which have the capacity to contribute to the survival of cancer cells under stressful conditions. Therefore, this area of research deserves more focused attention in the future to clearly assess whether the observed effects of MSC EVs are linked to the genetic alteration of cancer cells or, alternatively, if they are due to a ‘hit and run’ phenotype where EVs initially suppress tumor growth followed immediately by initiation of repair pathways which promote cellular migration and growth.

## Future Perspectives

The research outlined in this review highlights the multi-functional and “holistic” properties associated with stem cell EVs and how they may contribute to CNS repair. However, whether the primary modes of action are mediated by proteins, RNAs, or other EV-associated factors remains to be resolved. Therefore, further insight into the molecular and biochemical mechanisms by which stem cell EVs exert their functional effects is required, especially if EV-based therapeutics are to advance to a clinical setting. Equally significant from a regulatory perspective is immunogenicity. Along these lines, it is necessary to emphasize the observed hypoimmunogenic nature of stem cell EVs. As highlighted in a recent review, over 60% of the *in vivo* studies using MSC EVs have administered human material into various animal models with no adverse effects on immunogenicity (Elahi et al., 2020). Additionally, none of the research referenced in this review made specific references to any EV-associated toxicity.

Several other factors, including reproducibility, quality, and scalability must also be taken into account when considering the future of EV-based therapeutics (Phinney and Pittenger, 2017). These points could be addressed by the development of standardized protocols which would promote harmonization among laboratories and allow for tighter controls over the manufacturing and characterization of EVs. While there is no gold standard for EV purification, scalable technology, such as tangential flow filtration, is effective for the production of bulk material from a single donor and can also reduce lot-to-lot variability. Further purification can then be achieved through the use of immunoaffinity or ion-exchange chromatography (Colao et al., 2018). The use of these procedures could not only streamline EV manufacturing but also improve purity and yield as the use of chromatography offers the advantage of being able to isolate EV subpopulations based on their biochemical properties.

Recent advances in high-throughput technology have allowed for the unbiased screenings of the RNA and proteomic components of stem cell EVs and many studies have identified functionally relevant EV-associated cargo. As the field moves forward it will be critical to rigorously validate and confirm this data to achieve GMP compliance. Additionally, thorough characterization of the molecular content of each EV subpopulation (microvesicles, exosomes, exomeres, etc.) will be necessary to support the potential therapeutic use of stem cell EVs (Jafari et al., 2019). A more comprehensive assessment relating to the production of EV therapeutics has also been published by Gimona et al. (2017).

With respect to EV-associated cargo, it is important to acknowledge the recent work of others who have reported that media components often contain contaminating RNAs that co- precipitate with EVs. For instance, Mannerström et al. (2019) screened multiple samples of EV-depleted FBS as well as commercially manufactured serum- and xeno-free media. RNA sequencing data confirmed the presence of small ncRNA (e.g., miRNA, tRNA, yRNA, and snRNA) contaminants in each of these samples (Mannerström et al., 2019). In a similar study, Auber et al. (2019) also analyzed the RNA content of chemically defined, serum free media and found that exogenous miRNAs co-isolated with EVs. Interestingly, the supplemental component primarily responsible for this contamination was identified as catalase (Auber et al., 2019). This data underscores the absolute need for the inclusion of control samples of unconditioned media in order to properly validate all RNA sequencing data. From a regulatory perspective, this is a critical issue that should be addressed when considering the future therapeutic application of stem cell EVs. For a more thorough discussion of this topic we direct the reader to a recent review by Tosar et al. (2017).

## Concluding Remarks

There remains an unmet need for the development of effect CNS therapies. In recent years, the fields of EV research and stem cell biology have merged and, thus, paved the way for the development of next generation therapeutics which have the potential to simultaneously harness the regenerative properties of stem cells while reducing the potential adverse effects associated with stem cell therapy. Due to their ability to transfer a rich source of biological cargo between cells, EVs are natural mediators of cellular communication. As complex injury may necessitate a multi-faceted therapeutic approach, EVs may be naturally poised to deliver these effects. Relative to cell-based therapy EVs offer unique advantages including increased stability and potency, improved shelf life, and lower immunogenicity (Seo et al., 2019). The use of stem cell EVs therefore represents a new paradigm that holds tremendous potential for regenerative medicine. This holds true not only for the CNS, but for any pathological condition characterized by tissue injury or damage.

To underscore this, the COVID-19 outbreak can be used as an example. In the race to develop treatments against this novel infection in the face of a global pandemic, researchers and clinicians have turned to stem cells as an alternative therapeutic strategy. Two recent publications have already reported that transplantation of MSCs in critically-ill COVID-19 patients successfully reduced life-threatening symptoms and led to recovery (Leng et al., 2020; Liang et al., 2020). At the time of publication there were already over two dozen studies listed on clinicaltrials.gov that included stem cell therapy for COVID-19 treatment; one of which was specifically related to the use of MSC EVs. Additionally, our lab has begun testing the effects of stem cell EVs to repair COVID-19-related damage in multiple cell types (e.g., neurons, astrocytes, macrophages, and T-cells) that are important for regulating CNS function and BBB integrity. Now more than ever these factors provide a strong justification for the continued evaluation of EV-based therapeutics.

## Author Contributions

HB and FK wrote the manuscript. SP, DY, EA, MZ, NE-H, and LL aided in conceptual formation of the project, discussions, and editing. FK contributed to the overall direction and coordination of the review.

## Conflict of Interest

HB, SP, and DY are employed by ATCC and LL is affiliated with Ceres Nanosciences, Inc. The remaining authors declare that the research was conducted in the absence of any commercial or financial relationships that could be construed as a potential conflict of interest.
